# Efficacy and Implementation Planning Across the Veterans Affairs Polytrauma System of Care: Protocol for the REACH Intervention for Caregivers of Veterans and Service Members With Traumatic Brain Injury

**DOI:** 10.2196/57692

**Published:** 2024-08-15

**Authors:** Paul B Perrin, Jolie N Haun, Daniel W Klyce, Christine Melillo, Risa Nakase-Richardson, Ronald T Seel, Jennifer Martindale-Adams, Linda O Nichols, Robert A Perera, Bridget Xia, Bridget Hahm, Jeffrey Zuber

**Affiliations:** 1 School of Data Science University of Virginia Charlottesville, VA United States; 2 Central Virginia Veterans Affairs Health Care System Richmond, VA United States; 3 Department of Internal Medicine School of Medicine University of Utah Salt Lake City, UT United States; 4 James A. Haley Veterans' Hospital Tampa, FL United States; 5 Department of Physical Medicine & Rehabilitation School of Medicine Virginia Commonwealth University Richmond, VA United States; 6 Sheltering Arms Institute Richmond, VA United States; 7 Seattle-Denver Center of Innovation Rocky Mountain Regional VA Medical Center Aurora, VA United States; 8 Division of Pulmonary and Sleep Medicine Department of Internal Medicine University of South Florida Tampa, FL United States; 9 Department of Preventive Medicine University of Tennessee Health Science Center Memphis, TN United States; 10 Lt. Col. Luke Weathers, Jr. Veterans Affairs Medical Center Memphis, TN United States; 11 Department of Biostatistics School of Public Health Virginia Commonwealth University Richmond, VA United States; 12 Edward Hines, Jr. VA Hospital Hines, IL United States

**Keywords:** traumatic brain injury, telehealth, caregiver, methodology, veterans, service members

## Abstract

**Background:**

The responsibility of care for Veterans and Service Members (V/SMs) with traumatic brain injury (TBI) often defaults to informal family caregivers. Caregiving demands considerable knowledge, skill, and support to facilitate the health and well-being of V/SMs and themselves. Persistent and common TBI caregiver issues include strain, depression, and anxiety. While evidence-based, brief interventions have been developed and implemented for family caregivers in Veteran neurodegenerative populations, few interventions have been developed, adapted, or tested to support the unique needs of caregivers of V/SMs with TBI.

**Objective:**

This study will adapt and test an evidence-based, personalized, 6-session telehealth caregiver intervention, “Resources for Enhancing All Caregivers’ Health” (REACH), to meet the unique needs of caregivers of V/SMs with TBI. If successful, a community-based participatory research team will develop an implementation plan to roll out REACH TBI across the national Veterans Affairs Polytrauma System of Care.

**Methods:**

This mixed methods, crossover waitlist control clinical trial will use a Type 1 Hybrid Effectiveness-Implementation approach to adapt and then test the effects of REACH TBI on key TBI caregiver outcomes.

**Results:**

This study was funded by the Department of Defense in September 2023. Participant enrollment and data collection will begin in 2024.

**Conclusions:**

If effective, REACH TBI will be the first evidence-based intervention for caregivers of V/SMs with TBI that can be scaled to implement across the Veterans Affairs Polytrauma System of Care and fill a notable gap in clinical services.

**International Registered Report Identifier (IRRID):**

PRR1-10.2196/57692

## Introduction

Between 2000 and 2021, a total of 444,328 US military Service Members were diagnosed with traumatic brain injury (TBI) [1], leading to TBI’s designation as the “signature injury” of Operation Enduring Freedom (OEF), Operation Iraqi Freedom (OIF), and Operation New Dawn [2,3]. Between 7% and 23% of OEF/OIF/Operation New Dawn-era Veterans and Service Members (V/SMs) have experienced a TBI [2-5]. In 2009, the annual health care costs for OEF/OIF Veterans with TBI were almost 4 times higher than those without TBI (US $5831 vs US $1547), and the highest costs (US $7974) were shown in those with TBI, pain, and posttraumatic stress disorder (Taylor et al [5]). V/SMs receiving services in the Veterans Affairs (VA) have over 93,000 documented TBIs [6]. TBI can result in a constellation of long-term physical, cognitive, and neurobehavioral impairments [7-10]. Recovery time is variable, and many symptoms remain years or decades after injury, especially with more severe injuries [11]. As a result of these long-term consequences and care needs, rehabilitation medicine now unequivocally considers TBI a chronic health condition requiring long-term management and support [12].

Informal family TBI caregivers experience a myriad of unmet emotional, instrumental, and professional support needs regarding symptom management [13], and research has shown these high rates of unmet needs are closely associated with mental health problems in caregivers [14]. Caregivers often receive little formalized training or support in caregiving and symptom management, and physical symptoms in individuals with TBI are associated with greater family household needs, and emotional symptoms with greater family informational needs [15]. Among caregivers of V/SMs with TBI, over 40% of their needs go unmet with caregiver peer support, respite from caregiving, and help with caregiver negative emotions being the least often met needs; greater environmental barriers keeping the V/SM from participating in activities and the presence of V/SM mental health issues translate into more unmet emotional, community, and professional support needs [16]. Despite some parallels to civilian populations with TBI, V/SMs can have unique features of TBI (eg, polytraumatic and blast-related) and high rates of particular comorbid conditions (eg, posttraumatic stress and pain) that impact V/SMs’ health and needs [17-19] and may require additional and unique caregiving skills. Few military family members expect to provide the sort of long-term care that may be required by these complex injuries [20], and lack of training or formal support can compound caregiver strain and emotional distress [21]. Over time, military TBI caregivers experience declines in physical and mental health [22], reporting worse health than the general population [23]. Higher levels of TBI symptoms experienced by V/SMs are associated with greater caregiver strain and distress [24], which are in turn associated with caregiver grief and depression [24,25].

At present, there is no standardized, evidence-based, and widely implemented intervention for caregivers of V/SMs with TBI. Thus, there is a significant need for an evidence-based, portable caregiver telehealth intervention that is (1) adapted to TBI-specific needs, (2) relevant to caregivers of younger V/SMs with a range of neurobehavioral symptoms and strong potential for a positive recovery trajectory, and (3) readily accessible in rural and resource-limited communities. Resources for Enhancing All Caregivers’ Health (REACH) VA [26] is a successful evidence-based, 4- or 6-session telehealth behavioral intervention for caregivers and a VA national program for dementia [27], spinal cord injuries and disorders [28], multiple sclerosis [29], and posttraumatic stress disorder caregivers [30]. REACH has never been tailored specifically for or tested in caregivers of V/SMs with TBI. This study protocol describes the adaptation, evaluation, and implementation of a telehealth intervention, REACH TBI, for caregivers of V/SMs with TBI during the chronic phase of recovery.

## Methods

### Study Design

This study is a prospective mixed methods (greater emphasis on quantitative over qualitative data), type 1 Hybrid Effectiveness-Implementation study [31,32], with a crossover waitlist control clinical trial [33]. This is a multi-aim, multi-phase, VA-wide clinical trial that will include (1) engagement with caregivers of V/SMs with TBI and Polytrauma System of Care (PSC) clinicians to inform REACH adaptation for TBI, (2) a national waitlist control clinical trial, (3) development of an ambitious PSC implementation plan, and (4) a foundational community-based participatory research (CBPR) approach with stakeholders throughout every phase. Human-centered design (HCD) is a 5-step guiding framework integrating the study’s 3 aims and overall methodology and is fundamentally about identifying and responding to human needs [34]. As applied to implementation science in health care, studies based upon it often attempt to (1) develop an understanding of people and their needs, (2) engage stakeholders from early on and throughout the design process, and (3) adopt a systems approach to address systematical interactions among the micro, meso, and macro levels of health care [34]. During the initial HCD Discover and Define phases, interviews will be conducted with intended users and stakeholders (eg, TBI caregivers and PSC clinicians). These interviews will construct a narrative of TBI caregiver needs, engage intended users in meaningful discussion about REACH TBI appropriateness, and identify likely implementation facilitators and barriers. Ideas, emerging themes, and recommendations generated will help transition to the Design phase. The Design phase will inform REACH TBI optimization to meet the unique needs of caregivers of V/SMs with TBI. The Validate phase will test the effectiveness of REACH TBI nationally and support the Implementation phase when we develop an implementation plan to roll out REACH TBI across the PSC in a successive grant. As is consistent with the HCD, evaluation, progress, and milestone assessments will ensure process and outcome measures are met. The study is expected to recruit over 18 months.

### Setting

There are 4 research cores in this multicenter study. The University of Virginia (UVA; Charlottesville, Virginia) is the administrative core responsible for overseeing and directing the clinical trial in collaboration with multiple principal investigators leading the other 3 study cores. The James A. Haley Veterans’ Hospital (Tampa, Florida) is the data core where recruitment and data collection will be conducted. Virginia Commonwealth University (Richmond, Virginia) is the intervention core where the REACH TBI intervention will be delivered via telehealth. The University of Utah (Salt Lake City, Utah) is the implementation core where evidence-based strategies will be developed to implement REACH TBI throughout the VA PCS. Additionally, the University of Tennessee Health Science Center Caregiver Center (Memphis, Tennessee) will inform intervention protocol development and train REACH TBI interventionists.

### Intervention Development

Central to our team is the VA National Caregiver Center to leverage the foundational REACH [35] intervention—including its associated tools (eg, Caregiver Notebook and Risk Assessment), trainings, and delivery resources (Coach Manual [36] with scripts and checklists)—to accelerate a deployable REACH TBI protocol for clinical trial testing within 6 months of grant commencement. During the first 6 months of the grant timeline, adapting REACH for TBI will include extensive qualitative assessment involving CBPR engagement with subject matter experts (SMEs) and research team members throughout the PSC including caregivers of V/SMs with TBI, clinicians, clinical researchers, and administrators.

### Participants, Recruitment, and Sample Size

The eligibility criteria for V/SMs with TBI and caregivers are the following: (1) age of 18 years and older; (2) English-speaking; (3) primary caregiver for a V/SM who sustained a TBI at least 6 months prior; (4) primary caregiver who has provided care for a V/SM with TBI for at least 6 months; (5) provide some level of daily supervision or assistance with either a physical, cognitive, or behavioral issue they think is likely related to TBI; (6) believe that at least half of their caregiving responsibilities are likely related to TBI rather than another health condition or conditions; and (7) endorse a score of at least high burden (a score of 8 or higher) on the Zarit Burden Inventory-4 [37]. The exclusion criteria are (1) no access to the telephone or internet-accessible device, (2) auditory impairment that would make telephone use difficult, and (3) have completed a previous version of the REACH intervention. Participants had to report some level of burden associated with caregiving [35] based on prior research showing that burdened caregivers were more likely to benefit from the REACH intervention [38]. These criteria have been used successfully in caregiver studies to identify a stressed population.

A power analysis was completed to determine the number of participants necessary to achieve 80% power on the primary outcome assuming 10% attrition [39]. Baseline scores will be used as a covariate in the model as this increases statistical power, given that baseline and follow-up scores are usually highly correlated, resulting in a reduction in the error term of the model [40]. Assumptions used in computations included equal sample sizes in each contrast group, equal variances and attrition, probability of type I error of .05, and 2-sided testing of the null hypotheses [41]. Accordingly, 55 participants will be randomized to each group (N=110). With this sample size, we will have 80% power to detect a Cohen *d* of 0.50 assuming a correlation of pre- and postintervention scores of 0.50.

### Ethical Considerations

The UVA institutional review board (IRB) will be overseeing research activities carried out at all civilian research universities (UVA IRB-SBS Protocol # 6237). The University of South Florida (USF) is the reliance-agreement IRB of the James A. Haley Veterans’ Hospital and will be responsible for the oversight of the work occurring at this VA Medical Center (USF IRB Study # 006569). Using the VA’s corporate data warehouse, Veterans will be identified with a TBI diagnosis. Recruitment of their caregivers will be conducted remotely by research coordinators, who will screen potential participants for eligibility. Participants must give permission to be screened after hearing a study description that includes the components of informed consent including information about the study and the screening process itself. Study components will include a statement of the research (purpose, procedures), reasonably expected benefits to participants, and costs. The screening process components will include the duration of screening, alternatives, extent of confidentiality, and authorization for the release of protected health information for research purposes.

After the screening, consent forms, signed by the consenting research coordinator, will be emailed via secure Docusign (Docusign, Inc) or mailed with a postpaid return envelope, depending on the preferences of the caregiver. Informed consent will follow procedures of the UVA IRB (UVA IRB-SBS Protocol # 6237), USF IRB (USF IRB Study # 006569), and Research and Development Committee of the Tampa VA. Participants must give written or electronic informed consent before enrolling. During the informed consent call, the potential participant will be asked to read the informed consent and be given the opportunity to ask questions. A research coordinator will review all key aspects of the study with the potential participant and question to ascertain whether the potential participant has understood the information.

After consent is obtained and baseline data collected, participants will be randomly assigned to the immediate intervention group or waitlist control group. Each caregiver will be offered US $25 for each completed data collection and the exit interview for a total of US $100 (immediate intervention group) or US $125 (waitlist control group).

### Randomization

A block randomization schedule [42] (with 6 participants per block) will be created with a web-based computerized random number generator. The UVA research coordinator will maintain allocation concealment and eliminate possible selection or recruitment biases by keeping the randomization schedule concealed from the on-site research coordinator engaged in recruiting. The randomization schedule will be generated by the UVA research coordinator who will not have any contact with participants, and sequentially numbered sealed envelopes will be prepared prior to the recruitment of any participants. After the recruiting research coordinator determines eligibility for a prospective dyad and obtains informed consent, the UVA research coordinator will be notified and then open the next sealed envelope in the assignment sequence; the group assignment for that participant to one of the two groups will be revealed at that point. In this fashion, only the postdoctoral fellow interventionist assigned to provide REACH TBI will know the group assignment of a specific TBI caregiver. Because the intervention will be delivered by trained personnel who will have minimal contact with the waitlist control group (other than during the randomization call when the caregiver is informed of their study arm), we anticipate minimal overlap or “contamination” between these interventions and waitlist control group participants. Although participants and interventionists cannot be blinded during the study, all research staff involved in data collection and biostatistics staff involved in formal statistical analyses will be blinded to reduce bias and preconceptions in collecting and analyzing data.

### Intervention Implementation

The REACH TBI intervention will be carried out by a postdoctoral fellow interventionist who will be trained with didactic and hands-on content, knowledge assessment, skills practice, and role-playing for certification. Interventionist training includes strategies for overcoming problems associated with telephone interactions such as decreased cues and technological difficulties. The interventionist will use a mock caregiver to complete a role play of 2 key areas of the intervention: Target Concern Plan and Cognitive Reframing [43]. The Certification Role Play Observation Checklist for Individual Sessions used by the caregiver center will be used for the role play. The checklist includes behaviorally anchored ratings of specific procedural techniques (eg, correct use of forms) and clinical skills (eg, active listening). Performances will be observed for content and process. Feedback will be individually provided for each of the items listed on the checklist. The structure of the feedback will include positive behavior demonstrated; what behaviors should have occurred or occurred and were not in keeping with the protocol; and the rationale for the behavior that was expected. To assess intervention benefits accurately, early sessions for each interventionist will be monitored by study investigators, with caregiver permission. The investigators will provide feedback to the interventionist immediately after each session, focusing on fidelity, interventionist delivery, and evidence of caregiver receipt and enactment.

### Intervention

REACH TBI will be delivered by telephone in 6 individual hour-long sessions over 3 months, about every 2 weeks by a trained and certified interventionist. The REACH TBI sessions incorporate evidence-based components that have been shown to be crucial to successful caregiving interventions including problem-solving, cognitive reframing, and stress management [35]. The interventionist and caregiver negotiate the concerns to be addressed using those identified by the risk assessment [26,36]. Using problem-solving techniques, the interventionist and caregiver attempt to find effective and workable solutions to a specific target concern that is causing strain and stress for the caregiver, using the Caregiver Notebook. The target concern could be something related to the caregiver, such as asking family members for help, or to the care recipient such as bathing or driving. In this way, the intervention accommodates whatever concern the caregiver is experiencing—from activities of daily living or instrumental activities of daily living challenges to caregiver stress, guilt, or grief.

Each session is structured to build on the previous session using the protocol. Although tasks are structured and predetermined (eg, problem-solving), the focus of the task is a risk area (eg, safety), concern (eg, lack of support), or patient problem (eg, angry outbursts) that the caregiver has identified as troubling. One of the main foci of REACH is problem-solving. The interventionist teaches the ABC (Antecedent, Behavior, Consequences) method of problem-solving, and the caregiver and interventionist identify action-oriented behavioral strategies to address caregiving problems or V/SM behaviors using topics from the Caregiver Workbook in a Targeted Concern Plan. An outline of the intervention can be seen in Table 1.

**Table 1:** Outline of the REACH^a^ TBI^b^ intervention.

REACH TBI session or topic	Overview of content and structure
Assessment and session 1: stress management	Caregiver assessment Introduce intervention and review Caregiver Notebook Discuss stress Introduce stress management technique, signal breath
Session 2: problem-solving	Introduce session Review or modify the last session commitment, signal breath Provide general information about the Veteran or Service Member’s health condition Present safety material Introduce health care issues and health guide Problem-solve—target concern #1
Session 3: cognitive reframing	Review the health guide and safety Review or modify problem-solving plan #1 Make commitment for problem-solving plan #1 Introduce cognitive reframing
Session 4: problem-solving or cognitive reframing or stress management	Review the health guide and safety Determine caregiver goal attainment for cognitive reframing and review or modify, if needed Review or modify problem-solving plan #1 Determine caregiver goal attainment for problem-solving plans and review or modify, if needed If appropriate, identify target concern #2 Introduce problem-solving plan #2 or Work on cognitive reframing thought record
Session 5: problem-solving, cognitive reframing, stress management	Review the health guide and safety Determine caregiver goal attainment for any problem-solving plans and review or modify Review or modify cognitive reframing Offer stress management technique
Session 6 and Closure: problem-solving, stress management, and cognitive reframing review	Review Caregiver Notebook Review safety recommendations Review health and use of health guide Review caregiver well-being Review stress management techniques and strategies that worked Review cognitive reframing techniques Review problem-solving plans covered and strategies that worked

^a^REACH: Resources for Enhancing All Caregivers’ Health.

^b^TBI: traumatic brain injury.

### Data Collections

#### Quantitative Data Collection

**Figure 1:**
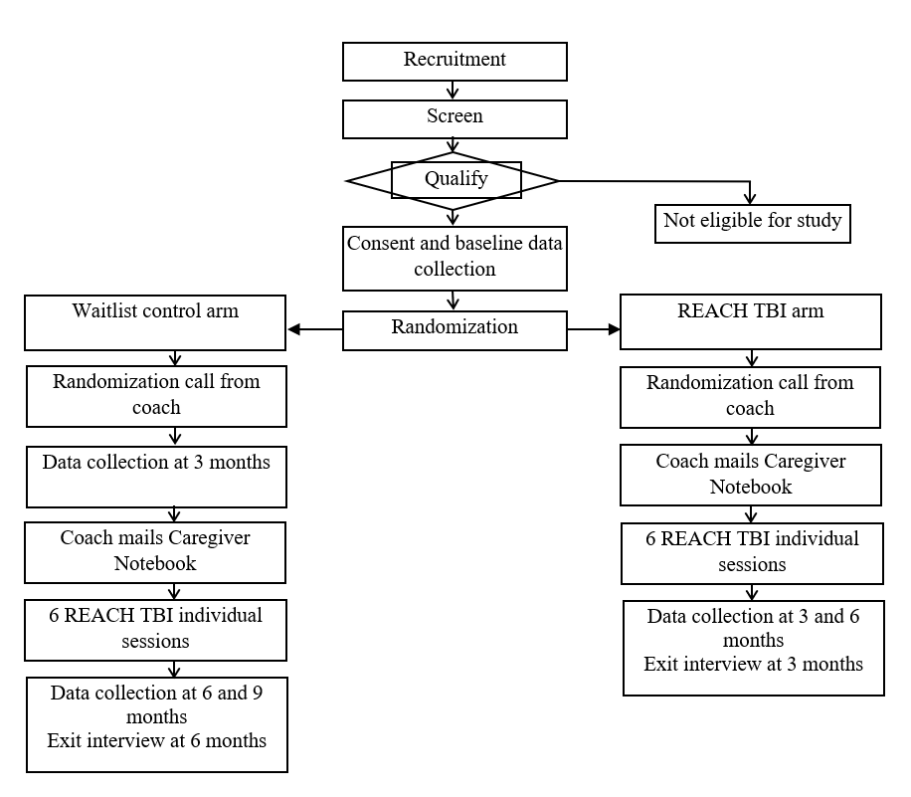
Waitlist control clinical trial timeline. REACH: Resources for Enhancing All Caregivers’ Health; TBI: traumatic brain injury.

Demographic and baseline data will be collected either by telephone or Qualtrics (Qualtrics International Inc) after enrollment and before randomization. Baseline data collection includes validated measures of key TBI caregiver outcomes such as strain, depression, anxiety, self-efficacy, and health care frustration. Follow-up data will be collected at 3 and 6 months from all participants and at 9 months from waitlist control participants using the same validated measures as during the baseline data collection. The study timeline is illustrated in Figure 1.

#### Qualitative Data Collection

A qualitative telephone exit interview will be conducted with all participants at the 3-month follow-up, after their last session, to evaluate their experiences using REACH TBI with a focus on caregiver satisfaction, usefulness, benefits, challenges, and implementation.

### Data Management

ID numbers will be used for all participants rather than participant names. Once analytic files are cleaned, all identifiable private information associated with an individual will be deleted from the analytic files, and analysis will be limited to data that are identified by an ID. Authorized project staff members will be assigned an active, unique user identification code and password to the server or network containing the data. The outcome measures will be compiled into a single survey format in Qualtrics and collected at baseline, 3, and 6 months from all participants and at 9 months from waitlist control participants.

### Outcome Measures

TBI Caregiver Quality of Life Caregiver Strain-short form 6a assesses caregiver strain [44]. A total of 6 items are scored from 1 (never) to 5 (always), with higher scores indicating greater strain. Among caregivers of V/SMs with TBI, internal consistency is α=.88, test-retest reliability is *r*=0.80, and convergent validity with mental health is *r*=–0.54 [45].

PROMIS (Patient-Reported Outcomes Measurement Information System) Emotional Distress—Depression—short form 8a assesses caregiver depression [46]. A total of 8 items are scored from 1 (never) to 5 (always). Scores range from 8 to 40 with higher scores indicating greater depression. Among caregivers of V/SMs with TBI, internal consistency is α=.94, test-retest reliability is *r*=0.70, and convergent validity with mental health is *r*=–0.73 [45].

PROMIS Emotional Distress—Anxiety—short form 8a assesses caregiver anxiety [46]. A total of 8 items are scored from 1 (never) to 5 (always). Scores range from 8 to 40 with higher scores indicating greater anxiety. Among caregivers of V/SMs with TBI, internal consistency is α=.89, test-retest reliability is *r*=0.65, and convergent validity with mental health is *r*=–0.66 [45].

PROMIS General Self-Efficacy assesses caregiver self-efficacy [47]. A total of 10 items are scored from 1 (I am not at all confident) to 5 (I am very confident), with higher scores indicating greater self-efficacy. Internal consistency is α=.94, and convergent validity with optimism is *r*=0.58 [47].

TBI-Caregiver Quality of Life Health Care Frustration—Self—short form 6a assesses frustrations a TBI caregiver has had with health care services [48]. A total of 6 items are scored from 1 (not at all) to 5 (very much), with higher scores indicating greater frustration with services received. Among caregivers of V/SMs with TBI, internal consistency is α=.96, test-retest reliability is *r*=0.89, and convergent validity with caregiver strain is *r*=0.60 [48].

### Data Analysis

#### Preliminary Analyses

Descriptive statistics will be computed for the total sample and stratified by treatment group for baseline participant characteristics. Means and SD (or medians and IQR) or counts and proportions will be calculated for continuous and categorical variables, respectively. In addition, an analysis of missing data will be conducted to examine factors that may be associated with the likelihood of missing data. Any factor found to be associated with the likelihood of missing data will be included in the final model.

Once assigned to a study arm, participants will be considered in the study, and we will follow an intention-to-treat approach (see below for details). Primary outcomes are measured for all participants at baseline, at 3 months, and after 6 months (or 9 months for the waitlist control group). We estimate the necessary sample size based on comparing groups at 3 months post randomization using baseline scores as a covariate.

#### Primary Quantitative Analyses

The intention-to-treat principle will be applied to all analyses. Once a participant is assigned to a treatment group, they will be included in the group in all analyses even if they end treatment or are lost to follow-up. Parallel analyses will be completed for each outcome. A linear mixed effects model [41] will be used to test the effectiveness of the REACH TBI intervention compared to a waitlist control initially for the primary outcome of caregiver strain. Contrasts will be coded to allow for tests of the mean difference between groups at 6 months postintervention and 3 months postintervention with 3-month measurements as the primary outcome. Each participant’s baseline score on the outcome will be included in the model in addition to a random intercept and fixed effects of treatment, time, and the treatment-by-time interaction. To account for multiple testing, Hochberg’s step-up procedure will be used to control the false discovery rate to .05. All analyses will be completed using the lme4 package [49] in the R statistical software (version 4.4.0; R Core Team).

#### Secondary Quantitative Analyses

Parallel analyses to those described in the primary analysis section will be completed for all secondary outcomes (eg, depression, anxiety, self-efficacy, and military health care frustration). In addition, group means will be compared at 6 months post randomization. Given that both groups will have been exposed to the intervention at this point, no difference between groups is expected. Finally, pairwise change within each group (eg, baseline vs 3 months and baseline vs 6 months within the control group) will be tested [41]. We expect that the treatment group will show improvement from baseline to 3 and 6 months post randomization but not between 3 and 6 months. For the control group, it is anticipated there will be no improvement or worsening between baseline and 3 months post randomization but will show improvement for comparisons of baseline and 3 months with 6 months post randomization.

#### Exploratory Quantitative Analyses

A moderation analysis [50] will be completed to examine how disability level and V/SM health conditions impact the effect of the intervention. Each variable and its interaction with treatment will be added separately to the linear mixed effects models described in the primary analyses section. If statistically significant interactions are found, results will be reported with the mean treatment effect, and ±1 SD on the moderator to improve results interpretability.

#### Qualitative Analyses

We will engage in content analysis [51] and use interview transcripts to sort descriptions, concepts, and central ideas into potential themes and concerns that occur repeatedly using the scrutiny techniques of repetitions and similarities and differences [52] and link themes to verbatim quotes [53]. Qualitative transcript data will be analyzed using descriptive content methods to identify domains or taxonomies about REACH TBI experiences. Content analysis will allow a priori coding framework derived from previous research literature and interview data to develop the coding scheme [51]. To adequately capture access determinants among participants, a qualitative codebook will be developed using deductive codes generated by construct relevance and inductively from interview data [54], as well as input from SME stakeholders (eg, TBI caregivers and PSC clinicians). Interview transcripts will be coded by the qualitative team using the codebook augmented by our SMEs to assess intervention satisfaction and implementation issues. Additional codes will emerge inductively from the interview data. The coding team will read 1 interview transcript separately and discuss the addition of new codes with examples. This process will continue with subsequent transcripts until no new codes are generated (code saturation) [55]. Intercoder reliability will be established when the coding team reaches at least 80% coding agreement [56]. Intercoder reliability will be routinely monitored to ensure consistency and limit potential drift in coding. Any discrepancies will be discussed and resolved among study staff during weekly meetings to ensure coding and analysis are completed on schedule or earlier. ATLAS.ti (version 22; ATLAS.ti Scientific Software Development GmbH), a qualitative analysis software program, will be used to manage and code interview text using a constant comparative approach. Coded text will be displayed in Excel (Microsoft Corp) spreadsheets to conduct a matrix analysis, a rapid assessment approach, which will be used to develop themes for the overall sample. Comparative matrices enable the identification of the most relevant, shared, and perhaps representative components, thereby enhancing the potential representation of the findings and allowing discernment of the most salient and representative experiences with REACH TBI identified by participants.

### Human Participants Protections and Adverse Events

We do not anticipate any severe adverse events or major psychological, legal, social, or economic risks from study participation. However, we anticipate the following (uncommon) minor risks: (1) participants may experience discomfort or fatigue in answering questions; (2) in the intervention, some of the discussion topics may be upsetting, and (3) if using a landline telephone, there will be no charges for calls from the REACH interventionist, but if using a cellular phone, calls by the REACH interventionist may use cellular minutes, and the participant may incur charges, depending on the user plan.

We will have multiple checks against adverse events. Alerts may be recognized during data collection, outside scheduled contacts, and during intervention sessions. Alerts or adverse event standardized procedures will address suicidal ideation, clinical depression levels, and safety risks. The procedure ensures that any alert is discussed immediately with a supervisor and appropriate action is taken. Alerts identified during data collection will be placed in the participant’s secure shared folder and an email sent to the interventionists and principal investigator (PI). Alerts will be discussed by the interventionist either during the randomization call for waitlist control participants or during the randomization call and session 1 for treatment-arm participants. The procedure ensures that any alert is discussed immediately, and appropriate action taken. For example, for clinical depression levels, we will follow up with the participant who will also be advised to contact their physician or other resource. All alert events will be recorded on the project alert form that includes the event date, whether the event is treatment-related, and the date the event was addressed. Interventionists will address alerts with the caregivers, complete the alert form, upload it to the participant’s secure shared folder, and email the PI that the alert was addressed. If appropriate, the PI will report the event to the IRB. Alerts, adverse events, and referrals will not cause a participant to be dropped from the study. Any contact outside data collection will be documented (time, reason, actions taken, and initiator) on an additional contact tracking form.

To ensure prompt reporting of research-related events, we will follow IRB guidelines. As soon as possible but in all cases within 1 to 5 working days, the PI will report to the IRB any changes to the protocol that were taken to eliminate hazards to a research participant, deviations to protect the physical well-being of a participant in an emergency, and any serious adverse event, related or possibly related to the research regardless of whether the event occurred during the study. Participants will have access to call study personnel and the PI during normal business hours to report concerning effects. A more thorough investigation of adverse effects will be performed as needed. While we do not anticipate serious adverse events that call for emergency care, if emergency care is required during a study visit, study staff will contact emergency services, and study staff will work with the participant to obtain such care. It will be emphasized to participants that they can take a break or discontinue either data collection or the REACH sessions at any time. An individual’s participation in the study will be terminated if the participant wishes to stop their participation in the study or in response to any significant adverse event determined by staff to warrant stopping study treatments. If the V/SM objects to their caregiver participating, this can also be a reason for discontinuation.

## Results

This study was funded by the Department of Defense in September 2023. Participant recruitment and data collection are expected to begin in 2024. Once REACH TBI effectiveness is demonstrated, the intervention will need to be spread throughout the VA PSC. Rigorous and systematic implementation planning will start in 2026 to broaden the impact of this clinical trial by rolling out REACH TBI among TBI clinicians across the PSC and sustaining intervention delivery.

## Discussion

### Principal Findings

Presently, there is no formalized or structured intervention for caregivers of V/SMs with TBI at a national level, and the existing services provided to caregivers for this population tend to be spread unevenly throughout the VA PSC. These gaps in services will be bridged by adapting the flagship program of the VA Caregiver Support Program’s National Caregiver Center—the evidence-based REACH intervention—to be responsive and relevant to the needs of caregivers of V/SMs with TBI. This intervention has the potential to serve the caregivers currently supporting the needs of nearly half a million V/SMs with TBI, remediating the adverse strain and mental health effects of caregiving, as well as improving self-efficacy and health care frustration. Supporting caregivers directly impacts V/SMs with TBI: higher caregiver health-related quality of life is associated with better functioning in V/SMs with TBI [57], and better caregiver outcomes directly impact the quality of informal care they can provide [58]. The REACH TBI intervention represents a substantial improvement over the current complete lack of a TBI-tailored, standardized but flexible, and evidence-based telehealth intervention available for caregivers of V/SMs with TBI. Further, the telehealth delivery format of the intervention makes it highly suitable for caregivers in rural or other resource-limited environments. This intervention has the potential to increase resilience within caregiving families [59], sustain gains made in functional recovery through the PSC, and ameliorate the negative impacts of TBI-related disability for caregivers.

We foresee several potential limitations and have proactively developed strategies and alternatives to address these. To ensure fidelity of treatment delivery across multiple interventions, the multiple principal investigators will be highly involved in training and supervision of the interventionists on an ongoing basis including regular meetings among these study team members and multiple checks on the reliability and fidelity of the treatment delivery. To support caregivers’ maximal engagement and retention in the trial, we will design the intervention to be responsive to new or ongoing emotional challenges among the participating caregivers, reasonably flexible to accommodate caregiver schedules and other life stressors or demands, and proactive in developing a structured retention plan. Finally, our team will be highly attuned to the complex needs of the V/SMs with TBI that might arise during the study, including detailed standard operating procedures to address behavioral health crises and referrals to appropriate services to address acute concerns within the VA Health Care System.

The near-term impact of this clinical trial will include multiple knowledge products advancing our options to prevent and treat complications resulting from TBI for V/SMs and their caregivers. The immediate outcome will include a manualized, highly portable intervention that can be delivered broadly by TBI clinicians. The intervention will include a resource companion, the REACH TBI Caregiver Notebook, which is being developed in consultation with subject matter experts in the areas of TBI, Military and Veteran health, and caregiving, as well as people with lived experience as TBI caregivers. This product will contain a wealth of information that will be relevant to caregivers of V/SMs regarding practical aspects of managing TBI, problem-solving, and reducing negative mood or affect. The long-term impact of this study is a standardized, evidence-based approach to supporting caregiving families for V/SMs with TBI throughout the entire PSC. This study will also catalyze a subsequent program of research focused on REACH TBI implementation (via subsequent grant proposals or formal PSC and Caregiver Support Program financial support to the caregiver center), to allow future evaluation of REACH TBI service utilization, cost, outcomes, and implementation. This study will provide a model for working from a CBPR framework to integrate perspectives of caregivers of V/SMs with TBI, clinicians who provide care to V/SMs with TBI, TBI clinical researchers, and PSC administrators in all phases of TBI intervention development and implementation.

Beyond its potential impact on the recovery and rehabilitation of V/SMs with a TBI and the well-being of caregiving military families, REACH TBI could be readily adapted and implemented in civilian health care systems. The modular nature of the training materials, the modifiability of the resource guide, and the digital platform to train a broad range of patient-facing staff throughout health care systems well positions REACH TBI to be used in a dual capacity among civilian populations.

### Conclusions

This protocol will use a CBPR methodology to fill a critical gap in meeting the complex needs of V/SMs with TBI and their caregivers. This protocol will target upstream factors affecting adjustment to TBI as a chronic condition, provide solutions to families and communities to mitigate the negative impacts of TBI on V/SM psychological health and functional outcomes, reach V/SMs and caregivers in resource-limited environments via a telehealth modality, and support the uptake of evidence-based interventions at enterprise scale. By engaging key stakeholders throughout the development, testing, and planning to implement REACH TBI, we expect that the resulting intervention will be highly relevant to the target population, usable to a range of providers in multiple settings, and scalable to disseminate broadly.

## Appendix

Multimedia Appendix 1 Peer review summary statement from the US Department of Defense.

## Abbreviations

CBPR: community-based participatory research
HCD: human-centered design
IRB: Institutional Review Board
OEF: Operation Enduring Freedom
OIF: Operation Iraqi Freedom
PI: principal investigator
PROMIS: Patient-Reported Outcomes Measurement Information System
PSC: Polytrauma System of Care
REACH: Resources for Enhancing All Caregivers’ Health
SME: subject matter expert
TBI: traumatic brain injury
USF: University of South Florida
UVA: University of Virginia
V/SM: Veterans and Service Members
VA: Veterans Affairs

## Data Availability

The data sets generated during or analyzed during this study are available from the corresponding author upon reasonable request.
