# Supplementary material for: Efficacy and Implementation Planning Across the Veterans Affairs Polytrauma System of Care: Protocol for the REACH Intervention for Caregivers of Veterans and Service Members With Traumatic Brain Injury
Source: JMIR Res Protoc. 2024 Aug 15;13:e57692. doi: 10.2196/57692 (PMC11362706; doi:10.2196/57692)
Supplement: Multimedia Appendix 1 [file resprot_v13i1e57692_app1.pdf]

Department of Defense
U.S. Army Medical Research and Development Command
Congressionally Directed Medical Research Programs
Fiscal Year 2022 Traumatic Brain Injury and Psychological Health Research Program
Clinical Trial Award - Research Level 3
Peer Review Summary Statement

CDMRP Log Number: TP220091
Grants.gov ID Number: GRANT13765209
Review Panel: Clinical Trial - Mental Health
Discussion Period: 02/07/2023-02/08/2023

Project Duration: 48 months
Budget Requested: $4,778,800
Direct Costs: $3,999,497
Indirect Costs: $779,303

Title: The REACH Intervention for Caregivers of Veterans and Service Members With TBI: Efficacy and Implementation Planning Across the VA Polytrauma System of Care
Principal Investigator: Paul Perrin
Performing Organization: Virginia, University of
Contracting Organization: Virginia, University of

OVERVIEW

The Principal Investigator (PI) of this application proposes to adapt an evidence-based, personalized, 6-session telehealth caregiver intervention, Resources for Enhancing All Caregivers' Health (REACH), to meet the unique needs of caregivers of veterans/service members with TBI. The specific aims are (1) to adapt and standardize REACH-TBI based on the TBI research literature and interviews with TBI caregiver and clinician subject matter experts (SMEs) (engagement and adaptation), (2) to evaluate the effectiveness of REACH-TBI in improving caregiver strain, depression, anxiety, self-efficacy, and military health care frustration outcomes (randomized wait-list control trial), and (3) to develop a structured and actionable implementation plan for a subsequent rollout of REACH-TBI throughout the full VA polytrauma system of care (PSC) (implementation planning). A multiaim, multiphase, VA-wide randomized wait-list control crossover clinical trial will be performed. The intervention is a 6-session telehealth caregiver intervention. The projected results are that caregivers receiving REACH-TBI will show improved strain (primary outcome), depression, anxiety, self-efficacy, and military health care frustration (secondary outcomes) compared to those in the wait-list control.

Procurement Sensitive Document
Do not copy or distribute without CDMRP written permission.
Prepared by SRA International, Inc., A GDIT Company, a support contractor to CDMRP

	Average Score	Standard Deviation
**Overall Evaluation**<br>*Rating Scale: 1.0 (highest merit) to 5.0 (lowest merit)*	1.5<br>**(Outstanding)**	0.2

**Criteria**<br>*Rating Scale: 10 (highest merit) to 1 (lowest merit)*	**Average Score**
**Research Strategy and Feasibility**	8.0
**Human Subject Recruitment**	9.0
**Intervention**	9.0
**Impact and Relevance to Military Health**	9.2
**Ethical Considerations**	10.0
**Statistical Plan and Data Analysis**	8.4
**Personnel and Communication**	9.3
**Community-Based Participatory Research**	8.8
**Data and Research Resources Sharing Plan**	8.5
**Transition Plan**	9.0

SCORED CRITERIA

Research Strategy and Feasibility
Average Score: 8.0

Scientist Reviewer A

This is a multiaim, multiphase, VA-wide randomized wait-list control crossover clinical trial. Specific aims are (1) to adapt and standardize REACH-TBI based on the TBI research literature and interviews with TBI caregiver and clinician SMEs (engagement and adaptation); (2) to evaluate the effectiveness of REACH-TBI in improving caregiver strain, depression, anxiety, self-efficacy, and military health care frustration outcomes (randomized wait-list control trial); and (3) to develop a structured and actionable implementation plan for a subsequent rollout of REACH-TBI throughout the full VA PSC (implementation planning). The objective is to adapt, rigorously test, and develop concrete plans to implement nationally across the VA PSC a previously developed 6-session telehealth caregiver intervention. Input will be sought from consumers, clinicians, researchers, and administrators regarding how the REACH intervention can be adapted to TBI. Regarding evaluation, 110 individuals will receive the intervention (ie, 6 one-hour interventions) over a 3 month period (the first 55 participants during Months 1 to 3, the second 55 participants during Months 4 to 6). Data collected will include baseline, intervention completion, and 3 month follow-up for each group and involve standard measures (ie, TBI quality of life, Patient-Reported Outcomes Measurement Information System [PROMIS] measures of mental health, subjective semistructured interviews). Final results will include the development of a plan to implement at a national level a proven intervention for caregivers of veterans and service members with TBI.

Strengths: The scientific rationale for the study is based on numerous similar studies that developed, evaluated, and implemented the REACH intervention to caregivers of military personnel with numerous other medical conditions (eg, dementia, multiple sclerosis [MS], amyotrophic lateral sclerosis [ALS], spinal cord injury [SCI], and posttraumatic stress disorder [PTSD]). The success of these previous studies suggests the likely success of a similar program for TBI. The request to develop a similar REACH-TBI

program is supported. The project timeline and feasibility appear appropriate given it is based on similar funded studies.

The study is well described and relatively simple in its design. The strength of the study is its foundation on numerous, previous studies with other medical populations. The current study is based on these others and uses similar methods, measures, and interventions except with caregivers with TBI. The initial part of the study will identify appropriate interventions for caregivers with TBI, the second part of the study will evaluate the effectiveness of this adapted REACH intervention, and the third part will develop and begin to implement the intervention to an extremely broad range of service providers who serve this population. The previously cited studies clearly indicate that the intervention leads to statistically and clinically significant improvements in health, functioning, and stress for caregivers of military personnel.

Stakeholder input is sought at all levels and across many sites and individuals (service members, veterans, researchers, clinicians, administrators). Their chosen measures are widely used across health outcome studies (ie, military and nonmilitary) and have proven to be very useful in previous studies (ie, ability to demonstrate improvement).

Weaknesses: The study proposes a crossover design in which 55 participants who receive the intervention are compared to 55 wait-listed controls over 3 months. Then the intervention and control groups are reversed for the next 3 months. This theoretically will allow for a comparison of the intervention to no intervention, but this assumes that the first intervention group will not demonstrate/report any benefits from the intervention when they serve as the control group (as is the case with crossover studies of medications). If the intervention does lead to lasting effects as proposed, then there are unlikely to be differences between the intervention and outcomes groups (the only likely difference will be between the first intervention and control groups at 3 months follow-up). A true control group is not addressed to determine if the intervention is effective or not (ie, sample with no intervention for 6 months, compared to 110 participants who receive the intervention).

The application is a bit inaccurate by stating the intervention involves “telehealth” when in fact it appears to involve telephone-based treatment. Without visual input available with telehealth, review of some of the intervention material will likely be less effective.

Scientist Reviewer B

Strengths: This is an area that is in need of interventions and prevention programs. The literature on caregiver mental health and strain is well reviewed, and a solid rationale for the study is provided. The research team currently has a grant that is looking at an adaptation of the REACH intervention for those with TBI and Alzheimer’s. Previous studies have also found versions of the REACH intervention helpful for dementia, spinal cord injury, MS, and PTSD with small to medium effects. The amount of data supporting this intervention is solid, although larger effect sizes would be ideal. Research level 3 is an appropriate research level requested for the intervention based on the presented data. The research team has shown the ability to run clinical trials of this size, and the current proposal is likely to be completed within the proposed period. The overall primary and secondary outcomes are appropriate. A huge strength of this study is the hybrid type 1 study methodology, which will allow for implementation barriers and outcomes to be studied as well. The qualitative and quantitative aspects of this study are complementary and useful to move the intervention to be ready for larger scale dissemination. This can help set up a longer-term plan (ie, the hybrid type 3 study mentioned as next steps). The initial REACH intervention used a community based participatory research (CBPR) approach, and the proposed study will continue with this. The integration and feedback of the stakeholder groups are important and integrated into the research process. Table 4 of the proposal does a nice job laying out potential problems and solutions to the major concerns. The research team has thought this through with multiple perspectives in mind.

Procurement Sensitive Document
Do not copy or distribute without CDMRP written permission.

Inclusion and exclusion criteria are appropriate and allow for a wide enough catchment of the target population. The instruments are common data elements (CDEs) and overall are well-established outcomes measures.

Weaknesses: The primary outcome of strain is not discussed enough from a data standpoint. It is a possibility that strain is a mechanism for some of the secondary outcomes (eg, mental health outcomes). However, this is not tested in the current study. Additional support for strain as a primary outcome is not developed to solidify the argument. The satisfaction and implementation interview will likely take more than 30 minutes and is not adapted to be shorter or to ensure the participants know the length may be extended. The sample will also consist of caregivers of veterans/service members who have a range of level of TBI including mild, moderate, and severe. Additional comparison groups are not integrated (eg, a support group or control that accounts for time and focus of intervention) as a third condition to understand if the REACH program is better than a more active control condition.

Human Subject Recruitment
Average Score: 9.0

Scientist Reviewer A

Strengths: The study has indicated that a sufficient number of participants will be recruited for the evaluation (n = 110), which the power analyses indicate will be appropriate to draw meaningful conclusions. In addition, the number of participants from which they can recruit is substantial and based on existing resources (eg, PSCs).

Weaknesses: The percentage of individuals from minority and underserved communities appears to be low (0/75 Native American, 1/75 Asian, 1/75 Pacific Islander). Information is provided about the racial/ethnic makeup of samples from previous REACH studies, but if they have such a large sample from which to draw they could increase the number of participants from underserved populations. Previous studies have recruited primarily women (70/75).

The study involves a crossover design, with 55 participants receiving the intervention initially followed by the 55 initial controls. This complicates the ability to determine the effects of the intervention to a true control group (ie, one that does not receive the intervention at any time). Identification of another control group that can be compared to the intervention group at 6 months post study is not developed that would allow for better generalization of the findings.

Scientist Reviewer B

Strengths: The study proposal will look at caregivers for primarily veterans who have a TBI. This population is one that the research team has area of expertise and access to within the VA system. Although some participants may be active duty, the majority will likely be veterans. The screening process and recruitment are strengths of this proposal. The team also has significant experience recruiting caregivers. Potential problems identified in terms of retaining and recruiting are addressed within the proposal in a meaningful way, including being flexible with scheduling. The proposal identifies that the majority of caregivers are women and so the majority of the sample will likely be women based on their previous work. The distribution of proposed enrollment is appropriate for recruiting more women than men given the previous data.

Weaknesses: The research does not include a plan to recruit unrepresented groups, which is an area of concern in this literature. The previous research by this group shows a somewhat diverse sample, with ~ 26% identifying as Black or African American, Asian, Native Hawaiian or other Pacific Islander, more

than one race, or unknown/not reported. Additional information on how to recruit from these groups is not developed.

Intervention
Average Score: 9.0

Scientist Reviewer A

Strengths: The intervention is strong as it is based on one that has been developed for service members and veterans with other medical conditions and shown to be effective for all. It is also very feasible and realistic as it is based on 6 one-hour interventions that are focused on problems identified by the caregiver and with manualized treatment interventions.

Weaknesses: The abstract and various parts of the application indicate that the intervention involves telehealth (implying video capabilities) when in fact it appears to involve only telephone-based interventions. Telehealth is increasingly used, particularly over telephone-based applications only, and has advantages in that visual information can be more easily shared and discussed, which may be important for any visual materials, notebooks, and manuals that may be shared.

Scientist Reviewer B

Strengths: The REACH intervention was developed by federal employees and is openly available for this trial. Supporting caregivers is an important area of work with significant data suggesting that the intervention is needed. Caregivers of veterans with other important issues (eg, dementia, PTSD) have a caregiver intervention, and this adaptation helps hit an important population. The previous clinical findings across caregiver programs that the current proposal would adapt are supportive of impacting the target outcomes and clinical need. The research components of this intervention are clearly defined in the consent form and processes laid out.

Weaknesses: It is not totally known what the correct dosing is of the intervention. This could be important to explore as the research team also identifies the time needed for the intervention and scheduling the intervention could be a barrier for some caregivers. Understanding what the potent dosage is could improve access to the intervention. The effect sizes for the interventions are small to medium on the primary outcomes. The 6 sessions may not be necessary for each caregiver. The intervention is still fairly resources intensive, which makes it unclear if this is sustainable longer term.

Impact and Relevance to Military Health
Average Score: 9.2

Scientist Reviewer A

Strengths: The study is directly related to improving the health and functioning of service members and veterans with TBI and their family members and is based on relevant research that indicates the need for increasing support for these populations given the increasing number who have experienced TBIs over the past decades and their impact on them and their families. The relevance is also demonstrated through a focus on use of telehealth/telephones to increase accessibility to service members in underserved areas (eg, rural US).

Weaknesses: No weaknesses were noted.

Procurement Sensitive Document
Do not copy or distribute without CDMRP written permission.

Scientist Reviewer B

Strengths: The results of this study could have a positive impact on caregivers, a population that is important to support. The trial focuses on strain and mental health outcomes, looking to see if the intervention has a positive impact on these outcomes as well as starting to understand the potential barriers to implementation of this intervention at a larger scale and across systems. The knowledge gained from this study could help understand how to best adapt REACH, determine if it works, and identify what might cause barriers to implementation—all of which are important to the psychological health for those impacted by TBI. If results turn out as planned, this project could have a long-term impact on caregivers, and this is a new intervention for caregivers that would be tailored using community based participatory research strategies. The research team has identified many issues that could come up and will also collect data on additional barriers to the intervention. Overall, the proposal is in an area of need.

Weaknesses: The program has been tested in several other areas and been shown to be effective. The current proposal does not focus enough on implementation of this across the VA system. The long-term impact of the intervention has not been studied with additional longitudinal data that would provide more insight into the long-term impact of the REACH intervention.

Consumer Reviewer

Strengths: This application intends to decrease the negative effects on caregivers for veterans and service members suffering from TBI by utilizing 6 telehealth sessions known as Resources for Enhancing All Caregiver’s Health (REACH). The success of this research would be moderately to greatly effective in providing caregivers the skills and resources to cope with negative effects of caring for a veteran or service member.

This application has the potential outcome to help the VA PSC further understand the needs and stressors of the caregiver as well as information to better provide applicable needed resources for the caregiver. This application has the potential to greatly impact the caregiver by documenting ongoing emotional and psychological markers given a particular caregiver’s individual resiliency. The success of this research may help decrease caregiver dependency on pharma by providing alternate proven mental health support and resources to the caregiver. This research helps fill a void in the documentation and assistance needed to help the caregiver of a veteran or service member with TBI. Over time, it’s inevitable that a veteran or service member’s condition will worsen and the demands on caregivers will increase. This anticipated increase in care directly correlates with added stress and negative impacts subjected on the caregiver.

Weaknesses: This application may not fully address the caregiver’s availability of time given other potential dependents such as children, daily responsibilities, or other constant stress of decreased governmental funding/removal of the caregiver program. This research only looks at a given period of time in a caregiver’s assistance to a veteran or service member suffering from TBI. Although the REACH program may provide some skills and resources to address the effects on a caregiver today, it may not be as impactful as a veteran or service member’s condition worsens over time. This research does not distinguish the severity of veteran or service member TBI patients participating in the study. This research may have skewed data points included in the outcome from possible distrust of the VA caregiver system and the potential for dismissal or decrease in benefits from showing improvements.

Discussion Notes

There was some discussion about study participants not being completely straightforward in answering questions due to a fear of dismissal or loss of benefits based on their answers, which could also apply to

Procurement Sensitive Document
Do not copy or distribute without CDMRP written permission.

service members once this is spread throughout the VA. This reduced enthusiasm for impact for some reviewers.

Ethical Considerations
Average Score: 10.0

Scientist Reviewer A

Strengths: As demonstrated through similar REACH research, all ethical obligations/considerations appear to have been met.

Weaknesses: No weaknesses were noted.

Scientist Reviewer B

Strengths: The impact of daily lives for caregivers is likely to be positively impacted including reducing strain and mental health. The overall population of caregivers is likely to benefit from this study to understand if REACH is effective. Additionally, the qualitative components will allow for caregivers and other stakeholders to be part of the research process and help shape the intervention and implementation strategy. The proposal has identified minimal risks as well as has safety plans in place to monitor and mitigate these risks. Participants will be given ID numbers and identifiable information will be deleted after data cleaning. Confidentiality and privacy policies are put into place to protect participants. Informed consent process is standardized and has been previously used.

Weaknesses: No weaknesses were noted.

Bioethicist Reviewer

Strengths: A strength is that the proposed trial might assist caregivers in caring for veterans and service members with TBI. A strength is that the knowledge to be gained could benefit caregivers for veterans and service members with TBI, the veterans and service members with TBI, and the VA system. A strength is that the level of risk is minimized by referring participants for treatment for major issues, by having investigators on call for participants, by assisting with accessing emergency care, by operating a standardized alert system, and by using a crossover wait-list control. A strength is that the study will employ appropriate safety monitoring and reporting for the minimal level of risk, including referring participants for treatment for major issues; by having investigators on call for participants; by assisting with accessing emergency care; and by operating a standardized alert system.

A strength is that privacy and confidentiality are appropriately considered by giving participants a unique identifier, maintaining identification links in a locked register, only disseminating deidentified data, and protecting stored data behind a secure firewall. A strength is that the process for seeking informed consent appropriately involves sending written information to potential participants and conducting a live screening and consent process via phone if the participant contacts the study. A strength is that exclusion and monitoring safeguards are in place for vulnerable populations.

Weaknesses: No weaknesses were noted.

Statistical Plan and Data Analysis
Average Score: 8.4

Procurement Sensitive Document
Do not copy or distribute without CDMRP written permission.

Scientist Reviewer A

Strengths: A strength of the planned analyses is their simplicity, i.e., demonstration of improvement in outcome measures compared to control group. The identified statisticians have worked on existing REACH studies and appear to have strong academic backgrounds, credentials, and track records.

Weaknesses: The proposal acknowledges weaknesses in not having a pure control group in that the initial intervention group is expected to demonstrate carryover effects, but the proposal then only indicates that "traditional carryover design statistical analysis (will not be) used." The specifics for how to address this weakness are not adequately expressed.

Scientist Reviewer B

Strengths: The statistical analyses will include a linear mixed-effects model, which is appropriate for the design. The intent to treat analysis is a strength given it is more conservative. Maximum likelihood estimation will be used for missing data, which is also appropriate. The exploratory moderation analysis by injury level is also an important one, although additional information on expected number of individuals within different levels of injury is not integrated. The qualitative data analysis will use an established software and a commonly used qualitative data analysis technique.

Weaknesses: Not enough details about the statistical plan (eg, linear mixed-effect model) are presented to evaluate the appropriateness. The power analysis uses a medium to large effect size, and the previous data suggest small to medium effect on the main outcome. It is unclear why this effect size was chosen given the presentation of the other data. Additional details (if comparisons will be made of other subgroups) are not developed.

Biostatistician Reviewer

Strengths: The design is a classic wait-list crossover design after randomization; one group will receive the 3-month intervention, while the other will wait 3 months and then start the intervention. Power is based on a difference between means and does embed this analysis in the repeated design. The analysis plan is appropriate for the design. Use of mixed models is appropriate to deal with attrition, repeated-measures analysis, and missing values, which are all discussed. The modeling process is discussed, including assessing interaction/moderation. Control for baseline and inclusion of covariates are discussed.

Weaknesses: Minor weaknesses are the following: Attrition is assumed equal in the 2 arms. There is no clear statement of the impact unequal attrition will have on generalizability and analysis. Missing values are assumed to be missing at random (MAR) or missing completely at random (MCAR), if the process leading to missingness is included in the model. While likely true, there is not a definitive statement for checking the assumption and a plan if the MAR assumption does not hold. Use of baseline Y is appropriate. However, which baseline to be employed for the wait-list group isn't specified. Likely, the baseline at 3 months is appropriate. Embedded in the analysis structure is a test of treatment x period interaction. If significant, while proper control for baseline may ameliorate some of the problem, there is a counterfactual issue.

Analysis of secondary variables is listed. A really small weakness is that if the primary hypothesis is not rejected, it is unclear how the results of these secondary analyses will be couched. And, if the primary does reject the null hypothesis, it is unclear whether there will be the same step-down procedures for testing of the interaction. It is not clear how the results will be assessed in the presence of a period x intervention interaction. No definitive statement for inclusion of time-varying covariates is presented. Incorporation of time-varying mediation (process) variables is not developed.

Personnel and Communication
Average Score: 9.3

Scientist Reviewer A

The PI, Dr Paul Perrin, is a professor of data science and psychology at University of Virginia (UVA) (Charlottesville, Virginia) and has a joint appointment as a research psychologist and codirector (with Dr Daniel Klyce) of the Polytrauma Rehabilitation Center (PRC) TBI Model Systems (TBIMS) Program at the Central Virginia (Richmond, Virginia) VA Health Care System. He earned a PhD in counseling psychology (2011) from the University of Florida (Gainesville, Florida).

Dr Christine Melillo is a health science specialist, nurse III at the James A. Haley Veterans' Hospital in Tampa, Florida. She earned a PhD in nursing (2018) from Uniformed Services University (Bethesda, Maryland).

Dr Risa Nakase-Richardson is professor in the College of Medicine, Department of Internal Medicine, University of South Florida (Tampa, Florida). She is the PI of the Tampa VA TBIMS (collaborating extensively with Dr Perrin and Dr Klyce, the MPIs of the Richmond VA TBIMS); senior research director at the TBI Center of Excellence at the Tampa VA; and the acting associate chief of staff for research at the Tampa VA. She earned a PhD in clinical psychology (2000) from West Virginia University (Morgantown, West Virginia).

Dr Daniel Klyce is an associate professor in the Department of Physical Medicine and Rehabilitation (PM&R) at the VCU School of Medicine and an MPI of the Richmond VA Polytrauma Rehabilitation Center's TBIMS study (with Dr Perrin, MPI). He earned his PhD in clinical psychology (2008) from Purdue University (West Lafayette, Indiana).

Dr Ronald Seel is professor and executive director of Center for Rehabilitation Science and Engineering (CERSE) at Virginia Commonwealth (VCU) School of Medicine (Richmond, Virginia), Department of PM&R. He earned his PhD in counseling psychology in 1999 from VCU.

Dr Perera is an associate professor and the graduate program director in the Department of Biostatistics at VCU, where he also has an affiliate faculty appointment with the Department of PM&R and serves as the lead biostatistician for CERSE. He earned his PhD in quantitative psychology (2013) from Notre Dame University (South Bend, Indiana).

Dr Haun has a joint, non-dual compensated, adjunct associate professor faculty appointment within the Division of Epidemiology in the Department of Internal Medicine at the University of Utah (Salt Lake City, Utah). Her PhD was earned in 2007 from the University of Florida in health, education, and behavior.

Dr Linda Nichols is a health services researcher at the Memphis VA Medical Center and professor, departments of preventive and internal medicine, University of Tennessee Health Sciences Center (UTHSC) (Memphis, Tennessee). She earned a PhD in anthropology in 1982 from Washington University (Saint Louis, Missouri).

Dr Jennifer Martindale-Adams is a professor, Department of Preventive Medicine, UTHSC, and a health services researcher at the Memphis VA Medical Center. She earned an EdD in counseling and personnel in 1993 from Memphis State University (Memphis, Tennessee).

Procurement Sensitive Document
Do not copy or distribute without CDMRP written permission.

Strengths: The proposed personnel are outstanding in their previous research to validate REACH interventions, as well as their overall research credentials. In addition, the study involves experts in TBI, health outcomes, and military and civilian populations. The ability to draw upon patient populations and extensive military programs is excellent (eg, PSC, VA Traumatic Brain Injury Model Systems [TBIMS], universities, Long-Term Impact of Military-Relevant Brain Injury Consortium [LIMBIC]). The research team is outstanding with numerous very successful researchers across numerous military programs (PSC, VA Caregiver Network) and universities (UVA, VCU). Their track record of successful completion of studies is excellent.

The study is being led by Dr Perrin with identified collaborators, including clinicians, researchers, and administrators from the PSCs, VA TBIMS, VA Memphis caregiver program, and multiple universities. The research team has an extensive, impressive record of research accomplishments and particularly with military populations and REACH interventions.

Dr Melillo has expertise in the complex intersections among stakeholder engagement, qualitative methods, quantitative data collection, and implementation science. She is a nurse researcher and has developed and published implementation tool kits for VA hospitals. Dr Seel brings extensive administrative leadership, database management, and research design expertise. Dr Klyce is a board-certified rehabilitation psychologist specializing in brain injury rehabilitation. Dr Haun is an implementation scientist with caregiver and TBI expertise, leadership, and training necessary to successfully support the proposed aims. Dr Nichols is a medical anthropologist and health services researcher with interests in family caregiving, including spousal and family caregivers of military personnel and veterans. Dr Martindale-Adams is a counselor, gerontologist, and health services researcher with interests in family caregiving and spousal and family caregivers of military personnel and veterans.

Weaknesses: No weaknesses were noted.

Scientist Reviewer B

Strengths: The study team composition is appropriate and has administrative and research support. Dr Perera is a biostatistician with significant experience on clinical trials. The research team brings together a variety of expertise that complements each other. The team members have worked together before and done similar level clinical trials including within the target population or similar populations. The communication plan and data transfer and management have been used before in other similar studies funded federally.

Weaknesses: The effort of the PI and other key personnel is not properly readjusted based on the year as different years appear to require different responsibilities as outlined within the proposal. Some effort levels are not clearly justified by the budget justifications.

Discussion Notes

Reviewers were enthusiastic about personnel with only minor concerns about some levels of effort not always matching the various responsibilities throughout the project.

Community-Based Participatory Research
Average Score: 8.8

Scientist Reviewer A

Strengths: The proposal involves the input of many stakeholders, including individuals with TBI and their caregivers from military samples. They also indicate a plan to share their findings with multiple communities. The current study also appears to have benefited from the participation of stakeholders in previous REACH studies.

Weaknesses: No weaknesses were noted.

Scientist Reviewer B

Strengths: The CBPR letters show strong support of the plan described within the proposal to have a variety of stakeholders integrated into the proposed research, including with the adaptation of the intervention to support its use within the proposed population. The CBPR plan includes stakeholder input across all aims of the study, which by achieving the aims focus on the adaptation, use of, and implementation plan for the REACH intervention. The plan also includes a dissemination plan for providing the results of the study to important caregiver communities/outlets.

Weaknesses: There are not enough caregivers represented within the proposed study. Although clinicians are important as they are delivering the intervention, caregivers will be receiving this and being able to disseminate may take a trusted caregiver message (an empirical question).

Discussion Notes

Some reviewers thought that there are not enough caregivers included, although this was considered a minor weakness for CPBR for most reviewers.

Data and Research Resources Sharing Plan
Average Score: 8.5

Scientist Reviewer A

Strengths: Plans to share data are consistent with normal standards and will allow others to use the data as appropriately requested in the future (ie, Federal Interagency Traumatic Brain Injury Research informatics system [FITBIR]) with excellent resources for dissemination (eg, VA PSC, TBI model systems).

Weaknesses: No weaknesses were noted.

Scientist Reviewer B

Strengths: The data will be presented at standard scientific conferences and peer-reviewed publications. The data from said publications will be made available upon request. The proposal uses CDE for all outcomes. Publications will be made available easily to those wanting access. Data will be uploaded to FITBR.

Weaknesses: As described, only data from publications will be made available. This limits the use of the data for the larger outside research community.

Procurement Sensitive Document
Do not copy or distribute without CDMRP written permission.

Discussion Notes

A reviewer had a concern that there are not enough details in the data sharing plan beyond publications that reduced enthusiasm for data and research resources sharing plan. Most reviewers felt that this is a minor weakness.

Transition Plan
Average Score: 9.0

Scientist Reviewer A

Strengths: The implementation plan is strong as it is based on similar studies on REACH that have been conducted to date. The third part of the study involves working with existing military-based programs to implement the intervention so that different clinical staff can administer the REACH-TBI intervention to caregivers of service members with TBI. The extensive network of providers and resources is extraordinary, particularly the number of staff (180) already trained in REACH interventions at the Memphis VA Caregiver Network program (23 network sites, 89 teams, 39 points of contact).

Weaknesses: No weaknesses were noted.

Scientist Reviewer B

Strengths: The outcomes of this project not only will show if the adaptation of REACH works within this population but also needs of caregivers and potential barriers to implementation. This will be the foundation of future work to disseminate and implement this both in the VA and at other places. The knowledge product translation plan addresses many of the transition targets with the potential outcomes (Table 1). The results of this study will set up the research team for a hybrid type 3 study, the timeline is reasonable for funding, and there are identified mechanisms for funding. The research team is well poised to continue this line of work as they are established in this area of work. The overall milestones are achievable and realistic and are appropriate for the phase of development. REACH was developed by this research team and is openly available.

Weaknesses: There is not a specific cost analysis done to show how cost of this type of intervention might impact the actual implementation. Although this may be a research aim in a future study as well, it would be important to understand if this ultimately will be feasible within a system.

Technology Transfer Specialist Reviewer

Strengths: The proposed study aims to adopt and test an existing intervention for families and caregivers of veterans and service members that are caring for patients with traumatic brain injury. Previously a similar program, REACH, was used for caregivers of patients with other disorders. The intervention aims to support the caregivers and provide them with tools needed for a successful outcome. Further studies are contemplated in the event of successful adaptation of the program in partnership with the VA and the DOD. The group plans to make all of the knowledge and tools available to the community for free through the TBI Model Systems network and the Model Systems Knowledge Translation Center that has a robust web presence and procedures for developing, piloting, and disseminating knowledge translation products about TBI.

Weaknesses: Although the translational approach seems well thought through and will be leveraging existing intervention tools and partnerships, the fact that there isn’t a plan for wider audience and adaption is a potential weakness. The team is explicitly not planning any proprietary products, which will

Procurement Sensitive Document
Do not copy or distribute without CDMRP written permission.

possibly limit the ability of this group to deploy these tools beyond the military and the VA. Nevertheless, significant planning had been put in place for this approach.

Discussion Notes

Reviewers were enthusiastic about transition plan, with weaknesses considered to be minor.

UNSCORED CRITERIA

Environment

Scientist Reviewer A

The proposal has indicated an outstanding environment of collaborators, settings, and results.

Scientist Reviewer B

All environments are well-established institutions that have held high levels of grants including clinical trials. The James A. Haley VA is part of the national network of polytrauma rehabilitation centers and a Defense Health Agency TBI Center of Excellence (COE). All sites have adequate institutional support, space, and personnel to achieve the proposed trial. Each site also has support from their administration.

Budget

Scientist Reviewer A

The budget appears to be appropriate, although the limited sample size is notable (n = 110) for the amount of funding requested.

Scientist Reviewer B

The direct costs are within the allowable range.

Application Presentation

Scientist Reviewer A

The proposal is exceptionally well written, which is not surprising given the experience and success of the proposed research team. The study is scientifically sound, clinically appropriate, methodologically simple, involves an extensive network, and shows high likelihood of success.

Scientist Reviewer B

The application was clear, and tables were helpful in making points succinct and easy to read.

Procurement Sensitive Document
Do not copy or distribute without CDMRP written permission.
